# Role of Medaka (*Oryzias latipes*) *Foxo3* in Resistance to Nervous Necrosis Virus Infection

**DOI:** 10.3390/ani14111587

**Published:** 2024-05-27

**Authors:** Wen Li, Zhi Wang, Jingjie Liang, Bilin Xia, Ruoxue Chen, Tiansheng Chen

**Affiliations:** 1 State Key Laboratory of Mariculture Breeding, Fisheries College of Jimei University, Xiamen 361021, China; 202111908021@jmu.edu.cn (W.L.); 202261000039@jmu.edu.cn (J.L.); 202211710036@jmu.edu.cn (R.C.); 2 Engineering Research Center of the Modern Technology for Eel Industry, Xiamen 361021, China; 3 Key Laboratory of Healthy Mariculture for the East China Sea, Ministry of Agriculture and Rural Affairs, Xiamen 361021, China; 4 College of Fisheries, Huazhong Agricultural University, Wuhan 430070, China; luke@webmail.hzau.edu.cn (Z.W.); xblin@webmail.hzau.edu.cn (B.X.)

**Keywords:** *foxo3*, *Oryzias latipes*, CRISPR/Cas9, viral infection, innate immunity

## Abstract

**Simple Summary:**

In this study, we investigated the survival rate and the expression of interferon-related genes of medaka (*Oryzias latipes*) *foxo3* mutants compared to WT after RGNNV challenge. This will be used for the study of disease resistance immunity-related genes in teleost.

**Abstract:**

Upon encountering a virus, fish initiate an innate immune response, guided by IFNs. Foxo3 plays a part in the body’s immune response; however, its specific role in the IFN-guided immune response in fish is yet to be clarified. In this study, we characterized *foxo3* in Japanese medaka (*Oryzias latipes*) and examined its role in the IFN-dependent immune response upon infection with the RGNNV. The results show that the coding region of the medaka *foxo3* gene is 2007 base pairs long, encoding 668 amino acids, and possesses a typical forkhead protein family structural domain. The product of this gene shares high homology with foxo3 in other fish species and is widely expressed, especially in the brain, eyes, testes, and heart. Upon RGNNV infection, *foxo3*^−/−^ mutant larvae showed a lower mortality rate, and adults exhibited a significant reduction in virus replication. Moreover, the absence of *foxo3* expression led to an increase in the expression of *irf3*, and a decrease in the expression of other IFN-related genes such as *tbk1* and *mapk9*, implying that *foxo3* may function as a negative regulator in the antiviral signaling pathway. These findings provide crucial insights for disease-resistant breeding in the aquaculture industry.

## 1. Introduction

Viral diseases often cause significant losses in farmed fish species. Viral nervous necrosis (VNN) is one such viral disease, caused by the small RNA virus Nervous necrosis virus (NNV), which is mainly encoded by genes *RNA1* and *RNA2* [1,2]. NNV primarily affects the central nervous system of fish, with the host range of the infection including both marine and freshwater farmed fish [3,4,5,6]. The virus poses a significant threat to fry and juvenile fish, with mortality rates reaching up to 100% within a week during severe infections. It also has a high mortality rate in adult fish [7]. It has been reported that the newly discovered CMNV (covert mortality nodavirus) is a typical alphanovirus, a type of NNV, originated from shrimp, that could switch their hosts to marine fish [8] by cross-species transmission, and also naturally infects goldfish [9] and zebrafish [10,11].

When fish encounter viral attacks, they activate their immune response mechanisms, which include innate and adaptive immune responses [12]. In this innate immune response, interferons (IFNs) play a key role in combating viruses [13]. Research has found that after viral infection, the survival rate of grouper juveniles that have been injected with IFN significantly increases [14]. It is currently known that there are mainly two types of IFNs in fish, INF-I and INF-II [15,16], among which INF-I plays a decisive role in the process of resisting viruses [17,18]. IFN-I can inhibit the replication and spread of the virus by inducing the expression of Interferon regulatory factors (IRFs) such as irf3 and irf7 [19]. A study has reported that the Forkhead box O3 (foxo3) transcription factor is also involved in the immune response, and that foxo3 restricts the expression of Irf7 and thus hinders the production of IFN-I, which plays a different role in the immune response involving interferon [20].

*Foxo3* is a member of the Foxo family, a notable feature of which is that it has a forkhead DNA conservative binding structure domain (forkhead domain) composed of about 100 amino acid residues. This structure can bind to a specific area in the promoter of the target gene, thereby effectively regulating the transcription of the target gene [21]. *Foxo3* is widely distributed in various cells, and it plays an important role in immune response, biological development, reproduction, and metabolism [22]. Research reveals that *foxo3* plays a negative regulatory role in immune regulation, and the expression of *foxo3* suppresses the growth of lymphocytes. In mice, overexpression of *foxo3* triggers apoptosis of B cells [23] and inhibits the proliferation of T cells [24,25], while the absence of *foxo3* expression promotes the apoptosis of neutrophils, thereby enhancing immunity to inflammatory diseases [26]. In zebrafish and mice, *foxo3* can suppress the IFN-directed immune response by inhibiting *irf3* and *irf7*, further promoting the proliferation of the virus [20,27]. These research results suggest that *foxo3* may be a potential regulatory factor for antivirals, but related research in fish is rare and needs to be further verified.

The Japanese medaka (*Oryzias latipes*) is a species of the *Oryzias genus* in the *Oryzlatidae* family of the *Cyprinodontiformes* order. Medaka is characterized by its simple maintenance, high stress resistance, short maturity cycle, and strong reproductive capability. Its genome and transcriptome information have been extensively studied and documented, so it is often selected as a model fish for research [28,29]. Due to medaka’s high sensitivity to viral infections, it is frequently used as a subject for virology experiments [30]. To further understand the role of the *foxo3* gene in the innate immunity of fish against viruses, we used the CRISPR/Cas9 gene editing technique to obtain *foxo3* mutants in Medaka. Then, through infection experiments with the Red-Spotted Grouper nervous necrosis virus (RGNNV), we studied the phenotype and mechanism of this gene in the process of antiviral resistance. This research will provide a theoretical basis for the screening of disease-resistant genes in fish, and for the breeding of new fish varieties with strong resistance.

## 2. Material and Methods

### 2.1. Animals

The medaka Hd-rR strain was raised in a water recirculation system (Shanghai Haisheng Biotech Co., Ltd., Shanghai, China) at 28 °C, with a light cycle of 14 h of light and 10 h of darkness [31]. High-quality circulating water is essential for medaka farming, and the parameters were maintained as follows: salinity, 0‰; pH of 6.7; ammonia, <0.19 mg NH_4_^+^/L; nitrite, <0.1 mg NO_2_^−^/L; nitrate, <20 mg NO_3_^−^/L [32]. Embryos were incubated in a constant temperature and light incubator (Ningbo Laifu Technology Co., Ltd., Ningbo, China) [33]. After hatching, the fry was fed freshly hatched brine shrimp 2–3 times a day, and reached sexual maturity at around 3 months. All procedures complied with the protocol approved by the Animal Care and Use Committee of Jimei University (Xiamen, China). All institutional and national guidelines for the care and use of laboratory animals were followed.

### 2.2. Phylogenetic Analysis of Foxo3

Foxo3 protein sequences of Japanese medaka (*Oryzias latipes*), zebrafish (*Danio rerio*), tongue sole (*Cynoglossus semilaevis*), large yellow croaker (*Larimichthys crocea*), giant grouper (*Epinephelus lanceolatus*), ruddy duck (*Oxyura jamaicensis*), chicken (*Gallus gallus*), rock pigeon (*Columba livia*), human (*Homo sapiens*), mouse (*Mus musculus*), black rat (*Rattus rattus*), turquoise killifish (*Nothobranchius furzeri*) and pig (*Sus scrofa*) were downloaded from the NCBI website (https://www.ncbi.nlm.nih.gov/, accessed on 5 May 2022). The ClustalW method in MEGA11 software was used to perform multi-sequence alignment analysis of the proteins and a phylogenetic tree was built using the neighbor-joining method, with the bootstrap method parameter value set to 1000 [34]. The phylogenetic tree was beautified using https://www.phylopic.org/ (accessed on 10 May 2022) website and Adobe Illustrator 2020 software.

### 2.3. Identification of the Coding Sequence of Foxo3

The medaka *foxo3* (Gene ID: 100301621) genomic DNA sequence was retrieved from the NCBI website (https://www.ncbi.nlm.nih.gov/), and the primers *foxo3* coding sequence (CDS)-F/-R were designed (Table 1). Total RNA from medaka ovaries was extracted by the TRIzol (RNAiso Plus 9109, TaKaRa, Beijing, China) method, and the PrimeScript^TM^ II 1st strand cDNA Synthesis Kit (Takara.6210A, TaKaRa, Beijing, China) was selected for reverse-transcription synthesis of cDNA. The CDS sequence of *foxo3* was obtained by PCR amplification using Q5 high-fidelity DNA polymerase (M0491S, New England Biolabs, Beverly, MA, USA). The PCR programs were set as 95 °C for 1 min, followed by 35 cycles of 98 °C for 10 s, 60 °C for 30 s, and 72 °C for 10 min. The amplified PCR product was subjected to 1% agarose gel electrophoresis to determine the correct size of the fragment obtained. The PCR product was purified and recovered by Gel Extraction Kit (D2500-02, Omega Bio-Tek, Norcross, GA, USA). Target fragment was ligated into pMD18-T vector (D103A, TaKaRa, Beijing, China), which was then transformed into *Escherichia coli* DH5α competent cells (EC0112, Thermo Fisher Scientific, Waltham, MA, USA). These were grown into colonies by overnight cultivation in a 37 °C bacterial culture incubator (DNP-9052, Shanghai Jing Hong Laboratory Instrument Co., Ltd., Shanghai, China), and single colonies were picked and added into LB liquid medium containing Ampicillin resistance to form bacterial fluid. The positive PCR fluid was used as a template for PCR identification. The positive PCR products were sent to the company (Wuhan Tsingke Biotech Co., Ltd., Wuhan, Hubei, China) for sequencing, and the sequencing results confirm the complete sequence of the *foxo3* CDS.

### 2.4. RT-PCR and RT-qPCR

The age of Japanese medaka was 3 months old, and the tissues sampled were eye, brain, kidney, liver, gill, heart, spleen, ovary and testis. Total RNA of 2 μg from medaka tissues was extracted by the TRIzol (RNAiso Plus 9109, TaKaRa, Tokyo, Japan) method, and the PrimeScriptTM II 1st strand cDNA Synthesis Kit (Takara.6210A, TaKaRa, Japan) was selected for reverse transcription synthesis of cDNA. The RT-PCR reactions were performed on a ProFlex™ 3 × 32-well PCR Thermal Cycling System (4484073, Servicebio, Wuhan, China), and the programs were set as 65 °C for 5 min, then rapid cooling on ice, followed by 42 °C for 60 min, 95 °C for 5 min, then cooling on ice. RT-qPCRs used to detect gene expression, and the primer sequences were summarized in Table 1. The RT-qPCR reactions were performed on a Real-Time Fluorescent PCR Analyzer PCR Instrument (qTOWER3G IVD, Analytik Jena AG, Jena, Germany) with 20 μL of reaction mixture that included 0.8 μL of diluent cDNA, 10 μL 2 x ChamQ Universal SYBR qPCR Master Mix (Q71103AA, Vazyme, Nanjing, Jiangsu, USA), 0.4 μL of each primer (10 μM), 0.8 μL of diluent cDNA and 8.4 μL ddH2O. Reaction were incubated in a 96-well optical plate (PCR-9602W-NS, Servicebio, Wuhan, China) at 95 °C for 2 min, followed by 40 cycles of 95 °C for 15 s, 60 °C for 20 s, and 60 °C for 15 s. The relative expression level of the examined genes was normalized to that of *β-actin* and calculated with the 2^−∆∆CT^ method. All data are expressed as the means ± SEM from at least three sets of independent experiments.

### 2.5. Construction of Foxo3 Mutants

Guide RNA target sites were designed using the CCTop website (https://cctop.cos.uni-heidelberg.de:8043/, accessed on 3 May 2020) [35], namely target sites sg1, sg2, and sg3. Sg1 is on the positive strand of DNA double-strand, while sg2 and sg3 are on the negative strand (Table 2). After selecting the sgRNA (Single guide RNA) target sequence, PAM sequence was removed, and a T7 promoter sequence and a sgRNA scaffold primer amplification sequence was added at the 5′ end and the 3′ end of the target sequence, respectively, to form the upstream primer of sgRNA, i.e., 5′TGTAATACGACTCACTATAGG-(gRNA 20nt)-GTTTTAGAGCTAGAAAT 3′. The downstream primer comes from the universal primer gRNA-R of the template pMD19T-sgRNA [36] (Table 1). Double-stranded sgRNA was amplified by PCR using the pMD19-T sgRNA backbone plasmid as a template. The PCR programs were set as 95 °C for 30 s, followed by 34 cycles of 95 °C for 5 s, 60 °C for 30 s, and 72 °C for 10 s. PCR products were recovered by agarose gel electrophoresis using Gel Extraction Kit (D2500-02, Omega Bio-Tek, USA) liquid, and sgRNA was transcribed using the mMESSAGE mMACHINETM T7 transcript kit (AM1345, Thermo Fisher Scientific, USA) and purified using the LiCI precipitation method. A mixture of Cas9 (300 ng/μL) and sgRNA (50 ng/μL) was injected into 1-cell-stage medaka embryos after Cas9 mRNA purification preparation [37]. Injected portions of embryonic DNA were extracted 4 days post fertilization, and fragments containing the target site of *foxo3* were PCR amplified using the detection primers *foxo3* D-F/D-R (Table 1). The PCR products were sequenced and compared to the wild type sequence to identify the mutation.

Subsequently, the injected F0 embryos were raised to adults, DNA was extracted by clipping the caudal fin and PCR amplification of fragments containing the target site was performed to verify the mutation. Then, the F0 mutant adults were crossed with the wild-type to obtain the F1 generation of mutants, and self-crossing of the F1 generation with the same type of mutation was performed to obtain the F2 generation, and the F2 generation was screened using PCR and T7 Endonuclease I (EN303-01, Vazyme, Nanjing, China) endonuclease for the homozygotes in the F2 generation for experimental use [38].

### 2.6. RGNNV Infection Experiment and Viral Quantitative Analysis

Three days post-hatching fry of wild medaka, 15 tails per group were housed in a 35 mm culture dish with 4 mL of embryo culture solution. RGNNV, extracted from diseased red-spotted grouper in our laboratory, was sequenced following PCR amplification of the full length using conserved RGNNV primers. The sequence was confirmed to be RGNNV, which contains double-stranded *RNA1* (2.9 kb, NCBI sequence No. MZ053461) and *RNA2* (1.4 kb, NCBI sequence No. MN105076.1). Viral titer was determined in GE (Grouper embryonic) cells using the median tissue culture infectious dose (TCID50). The viral titer was determined using the Karber method. The LD50 in medaka fry was set at 2 × 10^7.875^ TCID50/mL and this viral concentration was used for the experimental infection. A blank control group (wild medaka larvae without RGNNV), a control group (wild medaka larvae with 100 μL RGNNV), and an experimental group (mutant larvae with 100 μL RGNNV) were established, 15 tails for each experimental group, and the experiment was repeated seven times. Mortality in each group was monitored and recorded every 12 h. Finally, mortality curves were plotted based on the number of deaths to determine the mortality rates of the mutant and wild-type.

Infection experiments on adult fish was conducted by microinjecting 25 μL of RGNNV into the cloaca of 10 wild-type adult fish (control group) and 10 mutant adult fish (experimental group). The number of females and males of medaka in each of the experimental and control groups were 5 tails at 3 months of age. Sampling was performed 2 weeks post-infection. Given that RGNNV primarily attacks neural tissue-dense brain and eye tissues, we selected four tissues including brain, eye, gill, and muscle for subsequent experiments. Primers specific for the RGNNV *RNA2* gene (RGNNV *RNA2* qRT-F/-R, Table 1) were used, and qRT-PCR was conducted to detect the relative replication amount of RGNNV virus in each tissue of both the mutant and wild-type, and *β-actin* was using as an internal reference gene.

### 2.7. Statistical Analysis

To identify the significant difference between the two groups, Student’s *t*-test was performed. Groups were compared using one-way analysis of variance (ANOVA), followed by Dunnett’s test for pairwise comparisons. All statistical analyses were performed using GraphPad Prism 9 software, with *p*-values < 0.05 considered statistically significant.

## 3. Results

### 3.1. Characterization of Foxo3 in Medaka

**Figure 1:**
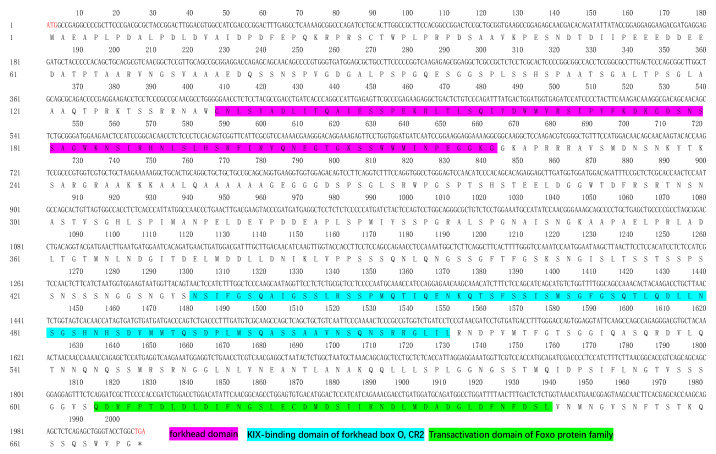
CDS and protein sequences of medaka (*Oryzias latipes*) *foxo3.* The foxo3 CDS sequence and the deduced protein sequences: purple: forkhead domain, blue: KIX-binding domain of forkhead box O, CR2 domain, green: Transactivation domain of foxo protein family domain. * represents the stop codon.

**Figure 2:**
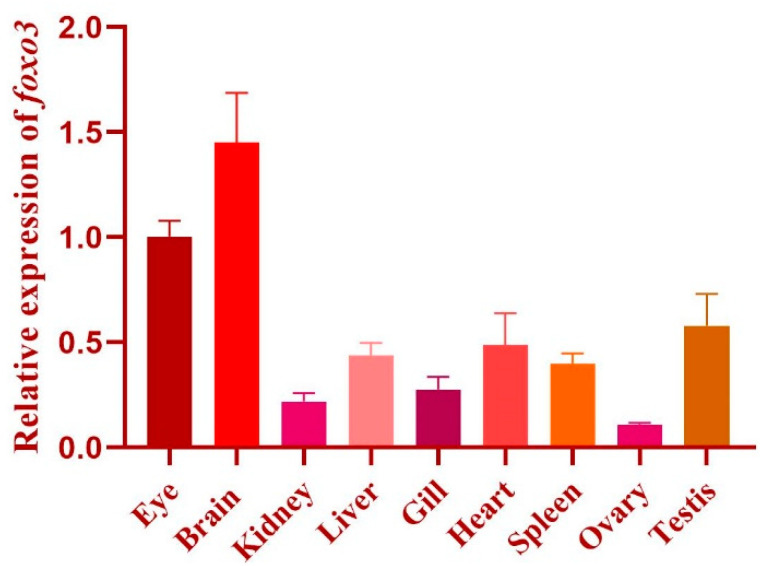
Expression pattern of *foxo3* in medaka adult tissues.

The structural analysis of the medaka *foxo3* gene indicates that it is located on chromosome 24, encompassing four exons and spanning a length of 2007 base pairs (bp) CDS. Exons 2 and 3 encode a protein consisting of 668 amino acids (Figure 1) and exhibit an anticipated molecular weight of 70.29 kDa. Further examination of the protein structure reveals three key structural domains within medaka foxo3: the forkhead domain (136–221 aa) from the forkhead family, the KIX-binding domain of forkhead box O (434–517 aa) from the CR2 family, and the Foxo Transactivation domain (605–646 aa). Expression analysis demonstrates that *foxo3* is expressed in multiple medaka tissues, including the brain, eyes, heart, gills, head kidney, liver, spleen, ovaries, and testes, with elevated expression levels observed in the brain, eyes, testes, and heart (Figure 2).

### 3.2. Phylogenetic Analyses of Foxo3

**Figure 3:**
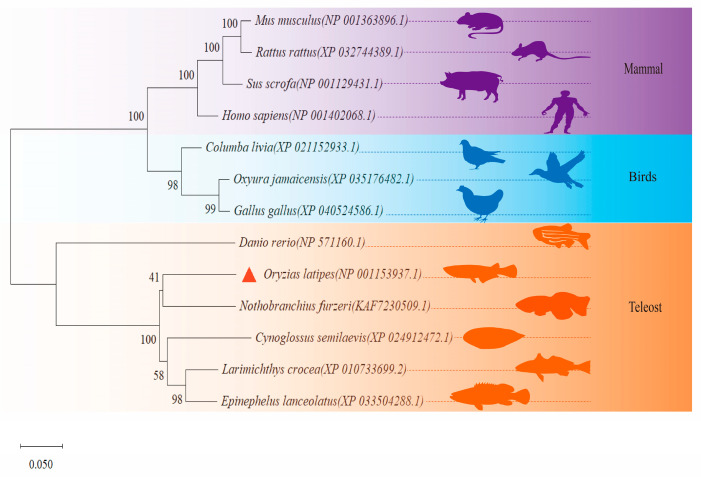
Phylogenetic tree of foxo3 protein in medaka and other species. The foxo3 of medaka is marked with a red triangle, and the areas in the evolutionary tree where the purple, blue, and orange backgrounds are located represent the clustering of mammals, birds, and fish, respectively.

Phylogenetic analysis results illustrate a division of the studied foxo3 proteins into three primary branches. The first branch comprises mice, black rat, pigs, and humans, denoting mammals. The second major cluster includes rock pigeon, ruddy duck, and chicken, signifying bird species. The third primary branch groups together zebrafish, Japanese medaka, turquoise killifish, tongue sole, large yellow croaker, and giant grouper, representing teleosts. Particularly within this teleost branch, the foxo3 of Japanese medaka exhibits a closer relation to the turquoise killifish, tongue sole, large yellow croaker, and giant grouper (Figure 3).

### 3.3. Construction of Foxo3 Mutant

**Figure 4:**
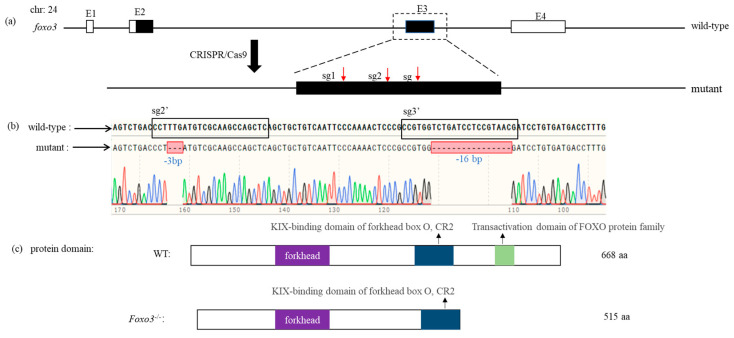
Comparison of DNA and protein domains in knockout strains of Medaka foxo3. (**a**) The medaka *foxo3* gene is 34,622 bp in length and contains four exons, of which the coding regions are (E2, E3), encoding a total of 668 amino acids. Red arrows above exon 3 indicate the positions of the three target sites; (**b**) sequencing results of the pure mutant, where the blue font represents deletion. sg2′ and sg3′ black boxes are the corresponding sequences on the other strand of the DNA double strand where the target site and the PAM sequence are located, and the red boxes represent the mutant base situation; (**c**) comparison results of the protein structural domains between *foxo3*^−/−^ and WT.

To knock out the *foxo3* gene, we designed three sgRNA sites on the third exon of the medaka *foxo3*. These sites, named sg1, sg2, and sg3, were co-injected with Cas9 mRNA into one-cell stage embryos of wild-type medaka. Following several rounds of screening across generations, we successfully obtained homozygous *foxo3* mutants, termed as *foxo3*^−/−^. This medaka mutant exhibits a deletion of 3 bases (−3 bp) at the sg2 site and 16 bases (−16 bp) at the sg3 site of the *foxo3* gene. Following analysis of the encoded protein sequence, we found that the mutant foxo3 protein (515 aa) lacks the activation structural domain typical of the foxo protein family, but retains both the forkhead family structural domain and the CR2 family structural domain (Figure 4).

### 3.4. Loss of Foxo3 Enhanced the Resistance to RGNNV in Medaka

**Figure 5:**
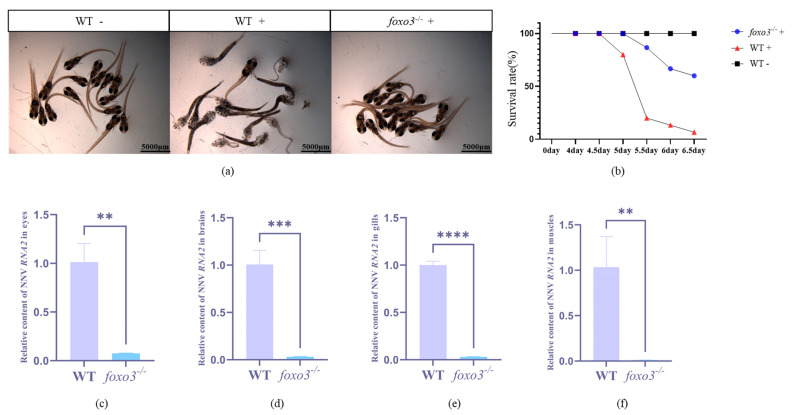
RGNNV challenge experiment of *foxo3*^−/−^ mutant larva and adult fish. (**a**) “WT” denotes wild-type larva, “*foxo3*^−/−^” denotes mutant larvae, “−” denotes no RGNNV virus added, “+” indicates plus RGNNV virus; (**b**) Survival curves of larvae, black squares represent wild-type larvae without viruses, red triangles represent wild-type larvae with viruses, and blue circles represent mutants with viruses; (**c**–**f**) The RGNNV virus replication ratios in 4 adult tissues (eye, brain, gill and muscle in order) of mutants and WT treated with RGNNV for 2 weeks. Error bars represent mean ± SD. ** *p* < 0.01, *** *p* < 0.001, **** *p* < 0.0001.

Infection experiments were conducted on *foxo3*^−/−^ fry and adult fish. The results demonstrated that the absence of *foxo3* significantly enhanced the resistance of mutant fry to RGNNV (Figure 5a,b). Following 6.5 days of RGNNV exposure, none of the 15 wild-type fry in the blank control group died, whereas in the RGNNV treatment experimental group, the mortality rate of wild-type fry reached 93%. Contrastingly, the mortality rate of mutant fry was only 40%. After the infection experiments on adults, we measured the replication amount of the RGNNV virus across different tissues. The findings revealed an inhibition in the expression of RGNNV *RNA2* in the eyes (Figure 5c), brain (Figure 5d), gills (Figure 5e), and muscle tissues (Figure 5f) of the *foxo3*^−/−^ mutants, indicating a suppression in virus replication.

### 3.5. Detection of Interferon-Related Gene Expression after RGNNV Infection

**Figure 6:**
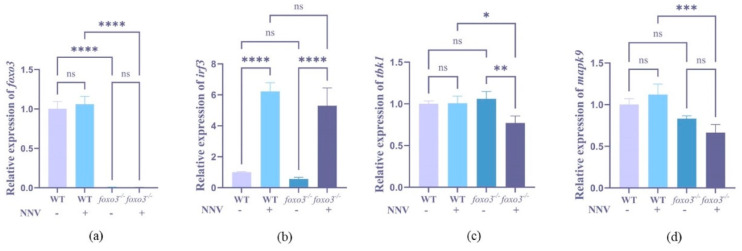
Relative expression of interferon-related genes in *foxo3*^−/−^ and WT. (**a**) Relative expression of *foxo3* in wild-type and *foxo3*^−/−^ induced by RGNNV. (**b**) Relative expression of *irf3* in wild-type and *foxo3*^−/−^ induced by RGNNV. (**c**) Relative expression of *tbk1* in wild-type and *foxo3*^−/−^ induced by RGNNV. (**d**) Relative expression of *mapk9* in wild-type and *foxo3*^−/−^ induced by RGNNV. Error bars represent mean ± SD. * *p* < 0.05, ** *p* < 0.01, *** *p* < 0.001, **** *p* < 0.0001, ns: no significance.

To elucidate the role of *foxo3* in the antiviral immune response, we examined the expression of IFN pathway-related genes (*foxo3*, *tbk1*, *mapk9*, *irf3*) in both mutant and wild-type fry under the influence of RGNNV virus. The expression of *foxo3* was notably reduced in *foxo3*^−/−^ mutants (*p* < 0.0001) (Figure 6a), which confirms the success of the knockout procedure. Upon induction with RGNNV, the expression levels of *tbk1* in *foxo3*^−/−^ showed a significant decrease (*p* < 0.001) (Figure 6c), and the expression of *mapk9* also diminished significantly compared to the wild-type medaka (*p* < 0.001) (Figure 6d). Conversely, the expression of *irf3* exhibited a significant increase post-virus induction (*p* < 0.0001) (Figure 6b). These findings collectively suggest that *foxo3* plays a role in the regulation of the IFN pathway.

## 4. Discussion

The Foxo family actively participates in various metabolic processes of organisms, including cell proliferation, differentiation, stress response, apoptosis, and immune regulation. Nevertheless, research into the influence of this family on the antiviral responses of teleost remains rare. In this study, we selected the Japanese medaka as our model organism and successfully constructed a *foxo3* knockout mutant using CRISPR/Cas9 gene editing technology. Upon conducting an RGNNV infection experiment, we observed a considerable increase in survival and a reduction in virus replication in *foxo3* mutants. Furthermore, we noticed a dysregulation in the expression of genes associated with the IFN pathway. Hence, our experimental findings propose that medaka *foxo3* potentially serves as an IFN-negative regulatory factor, playing an important role in medaka’s antiviral immunity.

The innate immune response, serving as the organism initial line of defense against viral invasion, can identify viral components and consequently producing pro-inflammatory factors and IFNs [39]. The IFN-related genes—*irf3*, *tbk1*, and *mapk9*—all exhibit responsive behaviors under viral stress.

*Irf3* serves as a key transcription factor, facilitating the synthesis of IFN-Ⅰ. In the face of RNA virus infections, its expression undergoes upregulation, which in turn enhances IFN expression and demonstrates antiviral capabilities [40,41]. Previous studies using zebrafish have shown that *foxo3* can reduce the expression of IFN by suppressing the transcription of *irf3* and *irf7*, acting as a negative regulator of the antiviral immune response [27]. In the present study, upon the knockout of *foxo3* in medaka, we also observed a significant upsurge in the expression level of *irf3* in the *foxo3*^−/−^ mutant infected by RGNNV. This indicates that this gene plays a significant role in the process of inhibiting virus replication and dissemination.

*tbk1* plays a vital role in the antiviral responses triggered by INF-Ⅰ, and its expression notably elevates when infected with RGNNV [42]. Furthermore, overexpression of *tbk1* can amplify the transcriptional activity of IFN promoters, which are regulated by *irf3* and *irf7*, subsequently leading to an increased expression of IFN [42]. Ubiquitin2 can further stimulate the induction of IFN-Ⅰ by phosphorylating *irf3* via *tbk1* [43]. In our study, we observed a significant decrease in the expression of *tbk1* in *foxo3*^−/−^ medaka compared to wild-type medaka. We hypothesize that this is due to the roles these genes play in the IFN-mediated signaling pathway where *tbk1* acts as a positive regulatory factor and *foxo3* serves as a negative regulatory factor. When there is a significant decrease in *foxo3* expression, IFN expression rises. This surge in IFN concentration likely triggers a negative feedback loop that affects the expression of *tbk1*, which promotes IFN, resulting in a downregulation of *tbk1*.

MAPK9, also known as C-Jun N-terminal kinase 2 (JNK2), is a member of the mitogen-activated protein kinase (MAPK) family [44]. This family plays a significant role in regulating both innate and adaptive immunity [45]. Studies in mice have demonstrated that JNK2 is required to produce IFN-γ [46,47]. Resveratrol (RES), an anti-inflammatory compound, can inhibit inflammatory responses induced by lipopolysaccharides (LPS). During LPS-induced inflammation, hyperactivation of the NF-κB and MAPK pathways, as well as stimulation of JNK phosphorylation, have been observed, suggesting an involvement of MAPK in LPS-induced inflammation [48]. Foxo3 can inhibit the activation of NF-kB [49] and block NF-κB/MAPK signal transduction to prevent ferroptosis, which may alleviate the symptoms of arthritis [50]. In this study, after the *foxo3*^−/−^ mutants were exposed to viral stress, the upregulation of the *irf3* gene could have triggered a substantial IFN production, inhibiting the excessive expression of *mapk9* and thereby promoting the survival of the fry. Consequently, the expression of *mapk9* in *foxo3*^−/−^ decreased.

## 5. Conclusions

In summary, this study analyses the molecular structure, expression pattern, and evolutionary lineage of the *foxo3* in medaka. Utilizing CRISPR/Cas9 gene editing technology, we have successfully engineered a *foxo3* gene knockout in medaka and concluded that *foxo3* could be potentially function as a negative feedback element during antiviral responses.

## Figures and Tables

**Table 1:** The primers used in the study.

Primer Names	Primer Sequence (5′-3′)	Size (bp)
*foxo3* CDS-F	TTTCAGGTGTCGTGAGGAATTCATGGCCGAGGCCCCGCTTCC	2007
*foxo3* CDS-R	AACATCGTATGGGTAGGATCCGCCAGGTACCCAGCTCTGAG	
*foxo3* RT-F	GACCTCCTCCCGCCGCAAC	179
*foxo3* RT-R	AGTTCTTCCATCCCGCAGAG	
gRNA-R	AAAAGCACCGACTCGGTGCC	20
*foxo3* D-F	ATGGACAGATTTCCGCTCTCG	832
*foxo3* D-R	AGCCAGAGTATTAGCCTCGTT	
NNV *RNA2* qRT-F	AAATGGTGGGAAAGCAGAACA	167
NNV *RNA2* qRT-R	CGAACACTCCAGCGACACA	
*foxo3* qRT-F	ACATCTTTCTCCAGCATCAGCA	180
*foxo3* qRT-R	GTTACGGAGGATCAGACCACG	
*tbk1* qRT-F	GCATCGGCGTTACCTTCTACCAC	156
*tbk1* qRT-R	CAATCTTGCCGTTTTCGCTCT	
*irf3* qRT-F	TCGACTCTGAAGCAGGAATCACA	151
*irf3* qRT-R	TCAGGCAACCCAGTTTCACCG	
*mapk9* qRT-F	CAGCCTCTGCCAGGTGATCCA	183
*mapk9* qRT-R	CTTGCCAGCCCAAAGTCCA	
*β-actin* qRT-F	TATCATTCGCCTGAAACCGAT	114
*β-actin* qRT-R	CTTTGCACATGCCAGATCCG	

**Table 2:** Target sites.

Target Site	Location	Forward and Backward	Target Sequence 5′-3′	PAM Sequence
sg1	24:7575244-7575266	+	GGTACGATGAACTTGAATGA	TGG
sg2	24:7575643-7575665	−	GAGCTGGCTTGCGACATCAA	AGG
sg3	24:7575693-7575715	−	CGTTACGGAGGATCAGACCA	CGG

## Data Availability

The datasets used or analyzed during the current study are available from the corresponding author upon reasonable request.
